# Optical mapping compendium of structural variants across global cattle breeds

**DOI:** 10.1038/s41597-022-01684-w

**Published:** 2022-10-13

**Authors:** A. Talenti, J. Powell, D. Wragg, M. Chepkwony, A. Fisch, B. R. Ferreira, M. E. Z. Mercadante, I. M. Santos, C. K. Ezeasor, E. T. Obishakin, D. Muhanguzi, W. Amanyire, I. Silwamba, J. B. Muma, G. Mainda, R. F. Kelly, P. Toye, T. Connelley, J. Prendergast

**Affiliations:** 1 The Roslin Institute, Royal (Dick) School of Veterinary Studies, University of Edinburgh, Easter Bush Campus, Midlothian, EH25 9RG United Kingdom; 2 Royal (Dick) School of Veterinary Studies, University of Edinburgh, Roslin, UK; 3 The International Livestock Research Institute, PO Box 30709, Nairobi, Kenya; 4 Centre for Tropical Livestock Genetics and Health, ILRI Kenya, Nairobi, 30709-00100 Kenya; 5 Ribeirão Preto College of Nursing, University of Sao Paulo, Ribeirão Preto, SP Brazil; 6 Institute of Animal Science, Agriculture Department of São Paulo Government, Sertãozinho, SP 14.174-000 Brazil; 7 Ribeirão Preto School of Medicine, University of São Paulo, Ribeirão Preto, SP 14049-900 Brazil; 8 Department of Veterinary Pathology and Microbiology, University of Nigeria, Nsukka, Enugu State Nigeria; 9 Biotechnology Division, National Veterinary Research Institute, Vom, Plateau State Nigeria; 10 Biomedical Research Centre, Ghent University Global Campus, Songdo, Incheon South Korea; 11 School of Biosecurity, Biotechnology and Laboratory Sciences (SBLS), College of Veterinary Medicine, Animal Resources and Biosecurity, Makerere University, P.O Box 7062, Kampala, Uganda; 12 Department of Disease Control, School of Veterinary Medicine, University of Zambia, P.O BOX 32379, Lusaka, Zambia; 13 Department of Laboratory and Diagnostics, Livestock Services Cooperative Society, P.O. BOX 32025, Lusaka, Zambia; 14 Department of Veterinary Services, Ministry of Fisheries and Livestock, Central Veterinary Research Institute, P.O. Box 33980, Lusaka, Zambia; 15 Centre for Tropical Livestock Genetics and Health, Easter Bush, Midlothian, EH25 9RG UK

**Keywords:** Genomics, Genome informatics

## Abstract

Structural variants (SV) have been linked to important bovine disease phenotypes, but due to the difficulty of their accurate detection with standard sequencing approaches, their role in shaping important traits across cattle breeds is largely unexplored. Optical mapping is an alternative approach for mapping SVs that has been shown to have higher sensitivity than DNA sequencing approaches. The aim of this project was to use optical mapping to develop a high-quality database of structural variation across cattle breeds from different geographical regions, to enable further study of SVs in cattle. To do this we generated 100X Bionano optical mapping data for 18 cattle of nine different ancestries, three continents and both cattle sub-species. In total we identified 13,457 SVs, of which 1,200 putatively overlap coding regions. This resource provides a high-quality set of optical mapping-based SV calls that can be used across studies, from validating DNA sequencing-based SV calls to prioritising candidate functional variants in genetic association studies and expanding our understanding of the role of SVs in cattle evolution.

## Abstract (summary)

**Table:** 

Measurement(s)	Optical Mapping
Technology Type(s)	Optical Mapping
Factor Type(s)	Structural variants
Sample Characteristic - Organism	Bos taurus
Sample Characteristic - Location	United Kingdom • Kenya • Zambia • Uganda • Brazil • Nigeria

## Background & Summary

Structural variants (SV) are a heterogeneous class of genetic variants involving large fragments of the genome (>50 bp)^1^. These variants include genomic insertions and deletions (InDels), inversions, duplications, translocations and more complex rearrangements^2^. Single nucleotide polymorphisms (SNPs) have been the primary focus of studies trying to map genetic loci underlying important cattle phenotypes. However, there are multiple lines of evidence suggesting SVs likely underlie many important cattle traits^3–6^. As many as 25–29% of all protein truncating events are thought to be caused by an SV in humans^1^ and notably, despite being less well studied, SVs have already been tied to key livestock phenotypes. For example, a duplication of the CIITA class II major histocompatibility complex transactivator gene in cattle has been tied to resistance to intestinal nematodes^7^ and a 12Kb copy number variant has been linked to mastitis in cattle^8^. Chromosomal translocations and duplications have been linked to skin pigmentation, a phenotype closely tied to environmental adaptation, and SVs across livestock species have been linked to phenotypes such as olfaction or resistance to adenocarcinoma-causing viruses^9^. Importantly SVs are responsible for approximately 5–10 times as many heritable nucleotide sequence differences between individuals than SNPs^10^. Unlike SNPs, that only effect a single basepair, and most often far from coding regions, SVs effect large regions and potentially multiple genes. Consequently, although smaller in number, any given novel SV event is more likely to have a phenotypic consequence.

The two most popular methods used to detect SVs are high-throughput sequencing (HTS) and array comparative genomic hybridisation (aCGH), both of which have been applied to European cattle^11–14^, but with few studies performed in other cattle breeds^15–17^. Each technology has advantages and limitations. aCGH involves measuring binding to probes covering the reference genome, and therefore it can only detect relative copy number changes between sample pairs and cannot for example detect novel insertions. Resolution is also limited. A major advantage of HTS approaches is that theoretically they can detect SVs at base-pair resolution. However, accurate calling of SVs from HTS data has proven to be difficult for a number of reasons including poor reference assemblies, chimeric reads, aligners penalising reads that don’t match the reference and the difficulties of sequencing and mapping to repetitive regions. This is exemplified by the generally poor agreement between SV callers even when run across the same samples^18,19^. Approaches using long reads and *de novo* assembly can still have true positive rates as low as 77%, even when using simulated data^20^.

Optical mapping (OM), a light microscope-based method that labels and physically locates specific motifs in the genome^21^, offers an alternative protocol to accurately detect large SVs. OM molecules can be consistently hundreds of Kb long, allowing for the detection of complex rearrangements undetectable using HTS. Despite the limitation of not being able to detect the actual sequence of the identified SVs, as well as missing smaller SVs, OM has a very high sensitivity and specificity, allowing for the generation of high-quality catalogues of SVs in individuals^22^. A study in humans successfully used OM reads to identify SVs in a total of 26 genomes revealing population-specific patterns of structural variation^23^.

In this study, we generated the first catalogue of cattle OM data for 18 animals from 9 different global breeds, and three continents, to better characterise common SVs across the cattle pan-genome. This data is a particularly valuable resource of SVs for the cattle species to intersect with other datasets, for example, for the validation of SV calls from other approaches^23,24^.

## Methods

### Sample preparation

We selected a set of 18 cattle across 9 divergent European, African and Indian breeds representative of Indicine, Sanga and Taurine ancestries (Table 1). Blood was collected by jugular venipuncture into EDTA vacutainers. Somatic recombination in B cells and T cells means the Ig and TCR loci in these cell types will be highly heterogenous, confounding accurate reconstruction of germline SVs at these loci from whole blood samples. Consequently, after the erthyrocyte lysis, monocytes were purified from the leukocytes using a MACS positive selection protocol with an anti-bovine SIRPα mono-clonal antibody (ILA-24^25^). Agarose plugs containing 5 × 10^5^–1 × 10^6^ of isolated monocytes were prepared using the Bionano Blood and cell culture DNA isolation kit (Bionano Genomics, San Diego, US) according to the manufacturer’s instructions and the extracted DNA used for analysis on the Bionano Saphyr platform to generate ~100X optical mapping coverage of each genome.

**Table 1:** Description of the samples.

Sampling Continent	Sampling Country	Group	Breed	ENA project ID	ENA sample ID
S. America	Brazil	Indicine	Nelore	PRJEB52551	ERS11891755
				PRJEB52551	ERS11891754
Africa	Kenya	Indicine	Boran	PRJEB52551	ERS11891767
				PRJEB52551	ERS11891766
Africa	Nigeria	Indicine	White Fulani	PRJEB52551	ERS11891768
				PRJEB52551	ERS11891769
Africa	Zambia	Indicine	Angoni	PRJEB52551	ERS11891764
				PRJEB52551	ERS11891765
Africa	Uganda	Sanga	Ankole	PRJEB52551	ERS11891756
				PRJEB52551	ERS11891757
Africa	Zambia	Taurine	Barotse	PRJEB52551	ERS11891762
				PRJEB52551	ERS11891763
Africa	Nigeria	Taurine	N’Dama	PRJEB47998	ERS8452869
				PRJEB47998	ERS8452868
Europe	United Kingdom	Taurine	Hereford	PRJEB52551	ERS11891760
				PRJEB52551	ERS11891761
Europe	United Kingdom	Taurine	Holstein-Friesian	PRJEB52551	ERS11891759
				PRJEB52551	ERS11891758

Table describing the breeds and ancestry of samples, with the continent and country of origin. The identifiers, as well as the ENA accession codes, for each of the two animals sampled per breed are also reported.

### Bionano Solve optical mapping processing

OM reads were filtered using the filter_SNR_dynamic.pl script with default parameters included with the Solve workflow, and then processed through the Bionano Solve^26^ pipeline (v3.3_10252018) using two different releases of RefAligner to overcome bugs preventing the successful assembly of the reads (version 7915.7989rel and 10330.10436rel). We generated the reference CMAP for the ARS-UCD1.2 genome with the Y chromosome from the 1000 bulls genome project (https://sites.ualberta.ca/~stothard/1000_bull_genomes/) using fa2cmap_multi_color.pl (default options and specifying DLE1 as the enzyme). The resulting data were imported into the Bionano Access (v1.6) software, and single-sample SVs were filtered using the recommended thresholds for SVs generated using Bionano Solve prior to v1.6.0 with the sizes recommended to achieve 90% sensitivity^27^: minimum insertion size of 5Kb, minimum deletion size of 5Kb, minimum inversion size of 100Kb, and minimum duplication size of 150 kb.

Filtered smap format files were converted to vcf format using smap_to_vcf_v2.py and sorted with bcftools (v1.10.2^28^). The resulting SVs were screened using bcftools and retained if 1) they had successfully been genotyped, 2) their size was >1Kb and 3) their quality was > = 20. The latter filtering largely removed all translocations, duplications, and complex events due to these having either very low (<1) or nil quality values.

We then defined the total amount of non-redundant reference sequence involved in a high-quality deletion. For each deletion, we calculated the central point in the genomic region affected by the SV:

$$Center=\frac{POS+abs\left(CIPOS\right)+END-abs(CIEND)}{2}$$

Where POS is the initial position, END is the end position, CIPOS is the confidence interval of POS and CIEND is the confidence intervals of END. Having defined the central point of the region, we defined the initial and final positions of the SV as:

$$BPI=Center-\frac{abs\left(SVLEN\right)}{2};BPE=Center+\frac{abs\left(SVLEN\right)}{2}$$

Where BPI and BPE are the limits of the SV and SVLEN is the size of the SV.

We then concatenated the regions for all the individuals, sorted them and merged them using bedtools sort and bedtools merge^29^ to remove any redundancies among the regions.

Following filtering, we merged the resulting variants within samples using SURVIVOR (v1.0.7^2^) accounting for the SV type and collapsing those whose break points were within 1 kb. We represented the intersection of SVs across individuals by extracting the support vectors generated by SURVIVOR^2^ at merging time, and plotted them using the UpSet function from the R^30^ package ComplexHeatmap^31^ (v2.8.0). We extracted the support value (i.e. how many animals present a specific SV) and SV size for each variant in the combined VCF and tested whether the SVs found in one individual only (support = 1) were significantly larger than those shared among individuals (support >1) by performing a Wilcoxon signed-rank test in a custom R script.

Finally, we defined which of the final set of SVs were found to potentially affect a gene. We ran VEP v105^32^ to predict which SVs were likely to disrupt a gene’s function, with the options --sift b (both preditions score and term), --nearest symbol (report the gene symbol), and --distance 200 (200 bp up and downstream consequence prediction). Those variants presenting coordinates referring to the negative strand (end position smaller than initial position) were manually fixed through an in-house script. We then investigated which SVs putatively overlap a coding region annotated in the cow genome by intersecting merged SVs with coding sequence intervals. Intersecting genes were investigated with FUMA^33^ to identify enriched gene ontologies and gene sets using all 35,142 gene elements with a unique Entrez gene ID as the background list.

## Data Records

The datasets presented here are stored at ENA under analysis IDs PRJEB47998^34^ and PRJEB52551^35^. The data are uploaded in Bionano BNX format compatible with downstream analyses. The output of the Solve workflows can be downloaded from Zenodo (10.5281/zenodo.6516993^36^ and 10.5281/zenodo.6517172^37^). The raw and filtered VCF files, converted using smap_2_vcf_v2.py, can be found on Zenodo with 10.5281/zenodo.6854879^38^.

## Technical Validation

### Assembly statistics and SV calling

We aligned the Saphyr optical mapping reads to the ARS-UCD1.2 genome^39^, expanded with the BTau5 Y chromosome generated by the 1000 bulls genome project, using Bionano Solve (v3.3 and 3.5) to assemble the genome maps and call SVs. The two NDama samples had previously been used to validate SVs using graph genome approaches^28^.

Workflow metrics are provided in Supplementary Table 1, summarising key metrics for the analysis of these samples in comparison to the recommended values from Bionano^40^.

Unfiltered molecules had average read lengths of 131.9–219.8 Kb (recommended >150 Kb) and molecule N50s ranged from 185.2–361.9 Kb across the samples (recommended >150 Kb). Following molecule filtering, all samples were within the recommended average length (245.5–383.1, recommended >230 Kb) and molecule N50 (245.0–426.5, recommended >230 Kb), and only 1 sample (Angoni 1) was slightly below the recommended label density (13.1–16.4, recommended 14–17). Importantly all samples passed the recommended values for the effective coverage of the reference (72.5–128.2, recommended >70) and of average confidence (30.1–43.2, recommended >20).

All samples also generated assemblies with high genome map N50s for both the diploid (71.7–85.0, recommended >50) and haploid (71.3–84.5, recommended >50) assemblies. Despite the low proportion of assembled contigs aligning to the reference genome (0.14–0.25, recommended >0.70), the high uniquely aligned length by reference length (0.835–0.906, recommended >0.85) shows the presence of long assembled contigs. The contigs present a high fraction of molecules aligned (0.77–0.94, recommended >0.80), effective coverage assembly (37.7–66.7, recommended >40) and average confidence (38.5–51.3, recommended >20).

Overall, 1 sample had 11 metrics within the recommended values, 6 had 12 metrics within the recommended values, 9 had 13 metrics within the recommended values and 2 had 14 metrics within the recommended values.

The Bionano Solve workflow identified a number of SV in each sample, ranging from 4,944 to 11,184 for a Hereford and Nelore, respectively (Tables 2). This mirrors the evolutionary distance of each sample from the reference genome, with the European taurine possessing fewer SVs (4,944–5,652) than the other samples, and an African taurine N’Dama possessing the least among the non-European individuals (N = 6,254). Relative SV numbers consequently broadly mirror prior expectations. Similar numbers of insertions and deletions were detected within each sample (insertion/deletion rate ranging between 0.966 and 1.065; Supplementary Table 1).

**Table 2:** Raw number of structural variants (SVs) and type detected in the different samples.

Sample	Deletions	Insertions	Duplications	Inversion breakpoints	Interchr. translocation breakpoints	Intrachr. translocation breakpoints	Total	Insertion/Deletion ratio
Angoni 1	4349	4505	45	91	13	4	9007	1.036
Angoni 2	4387	4673	64	100	11	7	9242	1.065
Ankole 1	4314	4324	67	111	10	8	8834	1.002
Ankole 2	3911	3984	66	101	8	5	8075	1.019
Barotse 1	3971	4044	42	52	11	6	8126	1.018
Barotse 2	4199	4159	67	106	7	9	8547	0.990
Boran 1	4935	5087	56	113	15	6	10212	1.031
Boran 2	4990	5007	68	138	6	14	10223	1.003
Hereford 1	2465	2380	43	41	11	4	4944	0.966
Hereford 2	2435	2437	77	88	9	7	5053	1.001
Holstein 1	2756	2759	48	52	15	18	5648	1.001
Holstein 2	2702	2801	59	76	9	5	5652	1.037
N’Dama 1	3411	3481	92	125	10	13	7132	1.021
N’Dama 2	3005	3082	67	86	6	8	6254	1.026
Nelore 2	5294	5508	58	113	11	5	10989	1.040
Nelore 1	5420	5499	96	136	15	18	11184	1.015
White Fulani 1	4467	4642	54	114	11	3	9291	1.039
White Fulani 2	4782	4805	41	45	17	14	9704	1.005

This table summarises the number of raw SVs detected in each sample, and their classification (e.g. insertion, deletion, duplication, inversion and inter- and intra-chromosomal translocation).

### Variant statistics

**Fig. 1:**
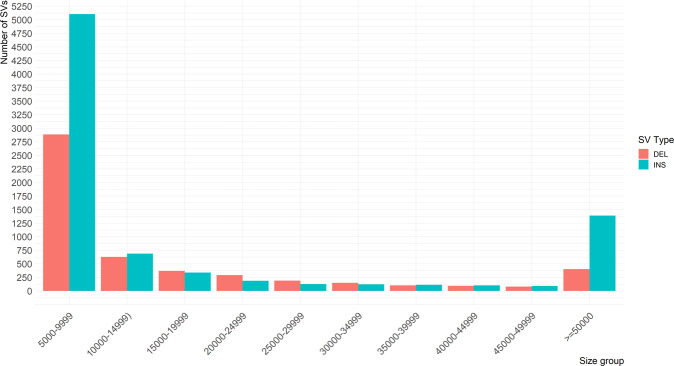
Histogram of the structural variant (SV) sizes. Histogram of the size of the identified SVs in bins of 5Kb.

SVs were filtered using Bionano Access, excluding SVs with unknown dosages, and retaining those larger than 1 Kb and with a quality > 20. SVs for each individual were then combined using SURVIVOR^2^ if the breakpoints were within 1 Kb, i.e. below the effective resolution of the approach^2^. This process allowed us to select a catalogue of 13,457 SVs across the genome, containing 8,262 insertions, 5,191 deletions and 4 inversions (see Supplementary Table 2 and 3 for the details on the type of SV identified). No duplications, inverted duplications and translocations passed the quality filtering. The imbalance in the number of insertions and deletions retained following filtering likely reflects the different sensitivity and specificity of optical mapping to detect the different classes of SVs^41^. The X chromosome appears to have more insertions than any other chromosome. This though is likely due partly to the difficulty of accurately calling SVs on the sex chromosomes, especially in males where effective coverage is halved (8 males among the samples). Further manual curation is therefore likely needed when working with the sex chromosomes. Consistent with results from previous studies^24^, most of the post-filtering insertions and deletions identified fell into the smaller classes, though 1,796 SVs (403 deletions, 1,389 insertions and 4 inversions) of over 50 Kb in length were identified (Fig. 1). While many SVs did not pass our stringent screening, they can still be recovered individually and included in future studies focusing on specific regions of the genome.

**Fig. 2:**
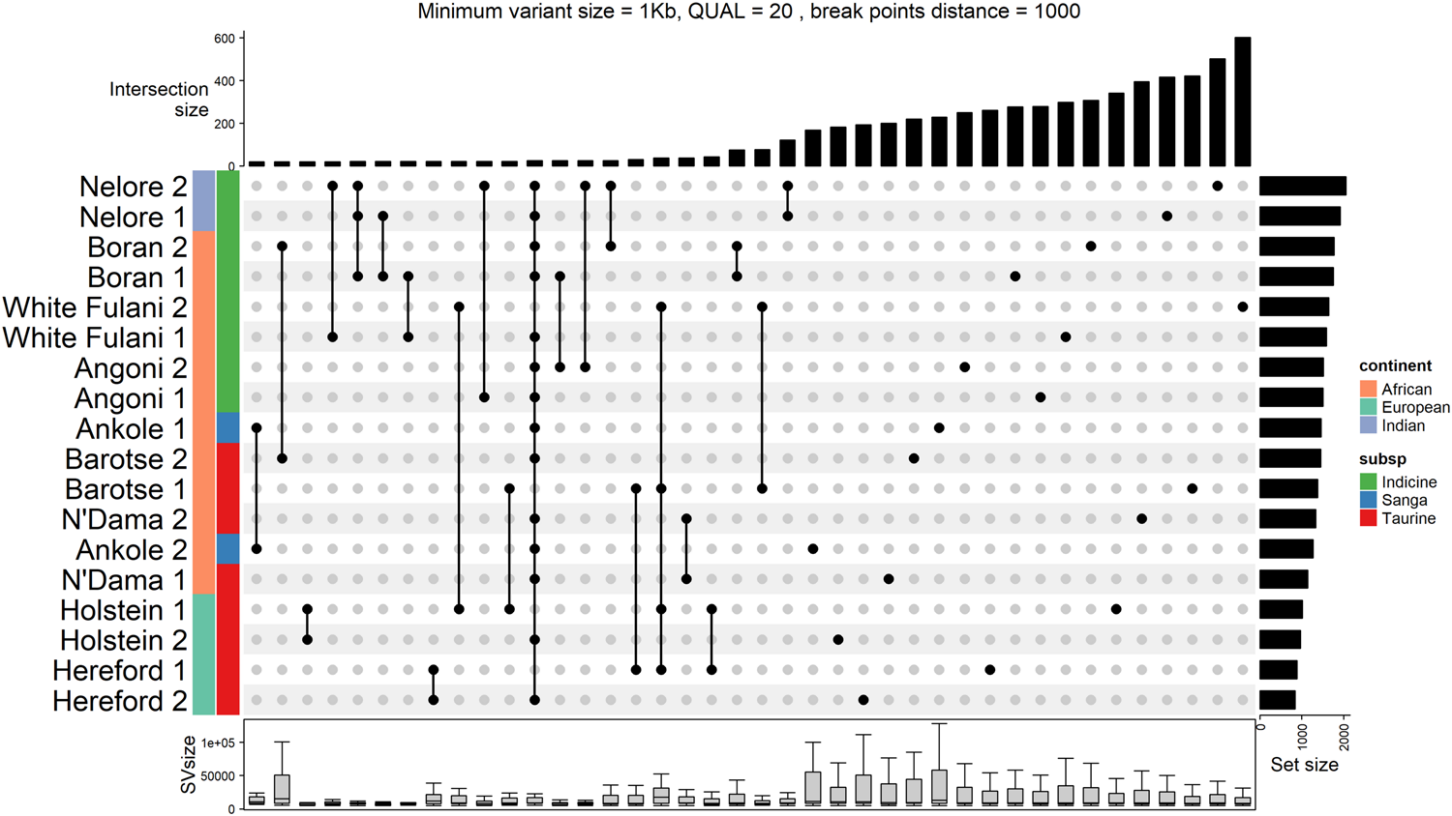
Upset plot of the structural variants. Upset plot of the structural variants by individual for the 40 sets containing the most SVs.

These SVs longer than 1 Kb and of high quality involve a total of 2,656 unique regions, for an estimated total of over 90 Mb of non-redundant bases (Supplementary Table 4). This number is comparable to what has been seen for novel sequences (i.e. insertions) using graph genome approaches, where an extra 70 Mb and 116 Mb of novel sequence were reported on 5 and 4 cattle reference genomes, respectively^24,42^. After merging the filtered variants from all the samples, most of the SVs were found to be private to an individual (Fig. 2), consistent with what has been observed in previous studies^1^. Individuals of indicine ancestry (Nelore and Boran) carry almost twice as many SVs relative to the Hereford reference as taurine individuals, further highlighting that the current reference poorly represents these breeds (Fig. 2).

**Fig. 3:**
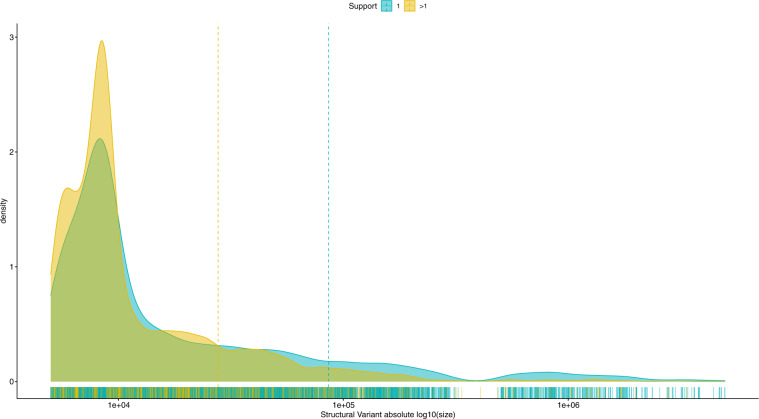
Density plot of the size of the structural variants found in only one (support = 1) or in more than one (support >1) sample. The strip of lines below the X axis shows the individual variant sizes, the vertical lines indicate the mean variant size, in each of the group.

Interestingly, we find that SVs only found in one animal (support = 1; n = 7,445, mean SV length = 85,954.23 bp) are generally larger (Wilcoxon rank-sum test P-value = 8.99 * 10^−37^) than the SVs found in more than one animal (support > 1; n = 6,012, mean SV length = 27,747.17 bp, Figs. 1, 3). The list of all SVs, with their position, support and size, are reported in Supplementary Table 5.

**Fig. 4:**
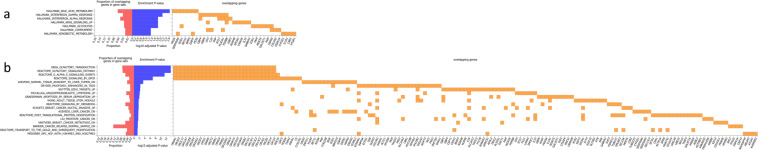
Gene set enrichment of genes potentially impacted by an SV. FUMA results showing the proportion of genes in sets, their enrichment and the heatmap of the genes in each for A) Hallmark gene sets and B) curated gene sets.

Finally, we investigated whether any of the high-quality SVs potentially impact annotated genes. VEP successfully processed 12,999 out of 13,457 SVs (see HTML report on GitHub). Some variants were too large to be successfully processed by VEP, and other were called as incomplete by VEP. Of these, 6,946 were intergenic, and the remaining 5,934 overlapped 5,780 genes and 17,386 transcripts, suggesting the potential for functional variants among the SVs detected. A total of 1,200 SVs putatively overlap a coding sequence. These coding sequences are included in a total of 884 unique gene elements in the cow annotation (Ensembl v105), and of these 483 have an associated gene name (Supplementary Table 6). A total of 292 out of 483 genes had an ID recognized by FUMA^33^. These 292 genes belong to a number of gene sets such as the Hallmark bile acid metabolism and interferon γ and α response sets (Fig. 4a), as well as the olfactory receptor curated gene set (Fig. 4b). All gene set results from FUMA are reported in Supplementary Table 7.

## Usage Notes

Even with the ever-decreasing cost of long read sequencing making it increasingly tractable to call SVs across sets of samples using HTS, validation of the SV calls remain challenging. This compendium of SVs across global cattle breeds provides a validation set called using an independent technology that can be used to assess the quality of cattle SV calls. In fact, optical mapping data has previously been used to validate sequencing based SV calls^24^, and we believe this dataset provides the largest set of optical maps to date for a livestock species.

With many SVs shared across the two animals of each breed, the raw molecules in this dataset can also be used to help scaffold and validate novel assemblies of cattle of breeds closely related to the individuals represented here, potentially reducing the cost of future genome assembly projects.

Unlike most cattle studies, this database is not focused just on European cattle breeds, meaning this will be a valuable resource to researchers across the globe. Importantly, it will allow for SVs to inform the interpretation of results from GWAS and population genetics studies by providing candidate functional variants in relevant regions.

Ultimately, we expect the database to enable further insights into SVs, an understudied class of genetic variation in cattle, giving access to a catalogue of thousands of variants present across multiple breeds worldwide.

## Supplementary information

Supplementary Table 2

Supplementary Table 3

Supplementary Table 4

Supplementary Table 5

Supplementary Table 6

Supplementary Table 7

Supplementary Information

Supplementary Table 1

## Data Availability

The code used in this article were deposited at https://github.com/evotools/CattleOManalyses.
