# Supplementary material for: Optical mapping compendium of structural variants across global cattle breeds
Source: Sci Data. 2022 Oct 13;9:618. doi: 10.1038/s41597-022-01684-w (PMC9561109; doi:10.1038/s41597-022-01684-w)
Supplement: Supplementary file 7 — Supplementary Information [file 41597_2022_1684_MOESM7_ESM.docx]

# Supplementary Material captions

The following supplementary materials are provided:

- Supplementary table 1 – Bionano solve quality metrics. This table provides the 15 different quality metrics generated by Bionano Solve for each of the samples analysed. Orange cells indicate those divergent from the recommended range of values whereas green cells indicate those falling within the specified ranges.
- Supplementary table 2 – Filtered number and type of structural variants (SVs) detected by class and size. Total number of SVs retained after combining the filtered variants for each sample in a single VCF, in each size class and for each type.
- Supplementary table 3 – Number of structural variants (SV) by type across the different chromosomes. The table shows the number of SVs on each chromosome for each type of variant, compared with the chromosomal length.
- Supplementary table 4 – Genomic regions from the ARS-UCD1.2 genome found to be putatively affected by a deletion. Each genomic region can encompass more than one individual deletion.
- Supplementary table 5 – Comprehensive list of the structural variants identified, with the size and number of individuals supporting the call.
- Supplementary table 6 – Genes found to overlap a structural variant identified in the 18 cattle.
- Supplementary table 7 – FUMA enrichment analyses of the 483 genes identified to be affected by a structural variant.
